# Quantitative Proteomics Reveals Common and Specific Responses of a Marine Diatom *Thalassiosira pseudonana* to Different Macronutrient Deficiencies

**DOI:** 10.3389/fmicb.2018.02761

**Published:** 2018-11-14

**Authors:** Xiao-Huang Chen, Yuan-Yuan Li, Hao Zhang, Jiu-Ling Liu, Zhang-Xian Xie, Lin Lin, Da-Zhi Wang

**Affiliations:** ^1^State Key Laboratory of Marine Environmental Science, College of the Environment and Ecology, Xiamen University, Xiamen, China; ^2^Key Laboratory of Marine Ecology and Environmental Sciences, Institute of Oceanology, Chinese Academy of Sciences, Qingdao, China

**Keywords:** marine diatom, *Thalassiosira pseudonana*, macronutrient, nitrogen, phosphorus, silicon, quantitative proteomics

## Abstract

Macronutrients such as nitrogen (N), phosphorus (P), and silicon (Si) are essential for the productivity and distribution of diatoms in the ocean. Responses of diatoms to a particular macronutrient deficiency have been investigated, however, we know little about their common or specific responses to different macronutrients. Here, we investigated the physiology and quantitative proteomics of a diatom *Thalassiosira pseudonana* grown in nutrient-replete, N-, P-, and Si-deficient conditions. Cell growth was ceased in all macronutrient deficient conditions while cell volume and cellular C content under P- and Si-deficiencies increased. Contents of chlorophyll a, protein and cellular N decreased in both N- and P-deficient cells but chlorophyll a and cellular N increased in the Si-deficient cells. Cellular P content increased under N- and Si-deficiencies. Proteins involved in carbon fixation and photorespiration were down-regulated under all macronutrient deficiencies while neutral lipid synthesis and carbohydrate accumulation were enhanced. Photosynthesis, chlorophyll biosynthesis, and protein biosynthesis were down-regulated in both N- and P-deficient cells, while Si transporters, light-harvesting complex proteins, chloroplastic ATP synthase, plastid transcription and protein synthesis were up-regulated in the Si-deficient cells. Our results provided insights into the common and specific responses of *T. pseudonana* to different macronutrient deficiencies and identified specific proteins potentially indicating a particular macronutrient deficiency.

## Introduction

Diatoms are the most diverse phytoplankton group in the ocean and are responsible for approximately 40% of total marine primary productivity (Nelson et al., 1995; Falkowski et al., 1998; Armbrust, 2009). They also play important roles in biogeochemical cycling of biogenic elements in the ocean. Macronutrients, such as nitrogen (N), phosphorus (P), and silicon (Si) are essential for growth, productivity and distribution of diatoms in the ocean (Dyhrman et al., 2012; Rosenwasser et al., 2014; Muhseen et al., 2015). Thus, adaptive ability to ambient macronutrient stresses is critical for the survival and proliferation of diatoms in the ocean.

Nitrogen is an important component of cellular structure and macromolecular compounds, such as proteins, nucleic acids, lipids, and pigments. Studies show that ambient N-deficiency affects N assimilation, carbon fixation, photosynthesis, pigment, and lipid accumulation of phytoplankton (Qian and Michael, 1993; Granum et al., 2009; Bender et al., 2014). Proteomic studies show that the metabolism of *Phaeodactylum tricornutum* shifts toward lipid accumulation rather than carbohydrate accumulation following N deprivation (Yang et al., 2014; Longworth et al., 2016). N stress triggers the accumulation of lipids through remodeling the intermediate metabolism rather than up-regulating fatty acid and lipid synthesis in *P. tricornutum* (Levitan et al., 2014). Transcriptional and metabolic results indicate molecular and metabolic modifications in the N-deprived cells (Alipanah et al., 2015). The response of central carbon metabolism under N starvation in *Thalassiosira pseudonana* differs from that in green algae and higher plants, and bears closer resemblance to the cyanobacteria (Hockin et al., 2012). N stress also impacts dimethylsulphoniopropionate synthesis (Kettles et al., 2014) and redox sensitivity (Rosenwasser et al., 2014). In addition, N sources and light exhibit a coupling effect on the urea cycle and N metabolism in *T. pseudonana* (Bender et al., 2012).

Phosphorus, as an essential nutrient for phytoplankton growth, participates in the formation of nucleic acids and membrane phospholipids, and regulates marine primary production (Dyhrman et al., 2007; Mather et al., 2008; Lomas et al., 2010). Studies show that P limitation drives the remodeling of membrane glycerolipid in diatoms (Martin et al., 2011; Abida et al., 2015). Transcriptomic and proteomic results demonstrate that *T. pseudonana* initiates multiple adaptive strategies, i.e., adjusting cellular P allocation and transport, utilizing dissolved organic P (DOP), regulating glycolysis and translation, and remodeling the cell surface in response to P-deficiency (Dyhrman et al., 2012). In *T. weissflogii*, P stress affects multiple macromolecular biosynthesis pathways (Wang et al., 2014). A recent study indicates that *Skeletonema costatum* can re-program its circadian clock and intracellular biological processes in response to ambient P-deficiency (Zhang et al., 2016).

Silicon is an essential element for diatoms to form their silica-based cell wall (frustule), which provides effective mechanical protection (Hamm et al., 2003). A set of genes involved in silica formation, signaling, trafficking, protein degradation, glycosylation, and transport are identified in *T. pseudonana* (Mock et al., 2007; Shrestha et al., 2012). Silicon transporters (SITs) are specific membrane proteins for silicic acid transport, and their mRNA and protein expressions and cellular uptake kinetics as well as localizations are characterized in diatoms (Thamatrakoln and Hildebrand, 2007; Sapriel et al., 2009; Shrestha and Hildebrand, 2015). These proteins are also involved in polyamine and cell wall synthesis (Frigeri et al., 2006). Si starvation stress affects Si transport, cell wall synthesis and cell-cycle progress (Du et al., 2014), resulting in lipid accumulation and gene expression changes in *T. pseudonana* (Smith et al., 2016).

The ecological success of diatoms suggests that they have developed a range of strategies to cope with various nutrient stress factors (Muhseen et al., 2015). It is of great interest to understand the adaptive responses of diatoms to different macronutrient stresses in the marine environment. Much effort has been devoted to the responses of diatoms to ambient macronutrient deficiencies, but these studies are mainly focused on a species under a particular macronutrient stress, and we know little about the common responses occurring during limitation for any macronutrient or any specific response occurring during limitation for a particular macronutrient. *T. pseudonana* is the first genome sequenced diatom species that provides a possible model for the study of response mechanisms of diatoms to ambient nutrient deficiency (Armbrust et al., 2004). In our study, we applied an iTRAQ-based quantitative proteomic approach to compare the protein expression profiles of *T. pseudonana* grown in nutrient-replete, and N-, P-, and Si-deficient conditions. The purpose of this study was to unveil common and specific responses of *T. pseudonana* to ambient N-, P-, and Si-deficiencies, and to mine specific proteins for indicating a particular macronutrient deficiency, so that we could gain insight into the global regulation of metabolic pathways in response to macronutrient deficiencies.

## Materials and Methods

### Culture Conditions and Sampling

A *T. pseudonana* CCMP 1335 strain was obtained from the Culture Collection Center of Marine Algae, Xiamen University, China. Cells were maintained in f/2 medium at a temperature of 20°C. A light intensity of approximately 100 μmol photons m^-2^ s^-1^ was provided by fluorescent lamps in a 14:10 h light: dark photoperiod. Four experimental groups were designed corresponding to four different nutrient conditions: a nutrient-replete culture with f/2 medium (the control), an N-deficient culture without N, a P-deficient culture without P, and a Si-deficient culture without Si. Each group was performed in triplicate.

Nutrient-replete cells were harvested at day 3 in the mid-exponential growth phase. The Si-deficient cells were collected at day 3, while the N- and P-deficient cells were collected at day 4. Cells were collected by filtering through a 2 μm pore-size membrane filter. The cells on the membrane were resuspended with sterile seawater, and then were centrifuged at 6,000 g for 5 min at 4°C. The pellets were collected for cellular elements, i.e., cellular C, N, P and Si, and macromolecular compounds, i.e., chlorophyll a, carbohydrate, protein and lipid as well as for quantitative proteomic analysis. The filtrates were collected to determine the concentrations of nitrate, phosphate and silicate.

### Physiological Parameter Analysis

Cell number, cell volume, and the maximum photosynthetic efficiency of Photosystem II (PS II; Fv/Fm) from each culture were monitored daily. Fv/Fm was determined using a Phyto-PAM Phytoplankton Analyzer (Walz, Germany). Cell number and cell volume were analyzed via a Z2 Coulter Counter (Beckman, United States).

Concentrations of nitrate, phosphate and silicate in the culture medium were measured using continuous flow analysis (CFA-SAN Plus/Skalar Analytik, Germany). Approximately 2 × 10^7^ cells per sample were collected for cellular carbon (C), N, P, and Si content analysis. Cells were transferred to the tinfoil cup, and were subsequently analyzed for cellular C and N contents using a vario EL cube (Elementar, Germany) after drying. Cellular P and Si contents were analyzed following procedures reported by Paasche (1973); Jeffries et al. (1979) and Boyd et al. (2016): cellular P was digested into dissolved inorganic phosphate using acidic potassium persulfate method and then calculated by measuring dissolved inorganic phosphate concentration using continuous flow analysis; cellular Si was digested by adding 0.2 M NaOH in a boiling water bath, and measured using continuous flow analysis after neutralization. Chlorophyll a was extracted using 90% (v/v) cold acetone and measured with a Turner Trilogy fluorometer (Turner, United States). Total carbohydrate (intracellular reducing pentoses and hexoses) content was measured using the anthrone method (Guerra et al., 2013). Total lipids were extracted using the modified method reported by Yoneda et al. (2018) and weighed on a microanalytical balance to calculate the crude content. Total protein content was measured as follows: cells were lysed in buffer containing 7 M urea, 2 M thiourea, 2% SDS, 40 mM Tris and 1% dithiothreitol with ultrasonic disruption, and then the total proteins were precipitated by adding threefold cold acetone at -20°C overnight. Protein was redissolved in lysis buffer containing 7 M urea, 2 M thiourea, 2% SDS and 40 mM Tris, and quantified using a 2D Quant kit (GE Healthcare, United States). For each physiological parameter, three biological replicates were analyzed. Statistical significance was analyzed using *T*-test.

### Protein Preparation and iTRAQ Labeling

A Sequential Extraction Kit (Cat No. 1632100, Bio-Rad, United States) was employed to extract proteins with different solubility. An appropriate amount of tributyl-phosphine was added to the reagent of the kit as the reducing agent. Cell pellets were first lysed in Reagent 1 containing 40 mM Tris with ultrasonic disruption. The precipitates were collected by centrifugation at 20,000 g for 10 min at 4°C and then lysed in Reagent 2 containing 8 M urea, 4% (w/v) CHAPS, 40 mM Tris and 0.2% (w/v) Bio-Lyte 3/10 ampholyte with ultrasonic disruption. Finally, the precipitates were collected and then lysed in Reagent 3 containing 5 M urea, 2 M thiourea, 2% (w/v) CHAPS, 2% (w/v) *N*-decyl-*N,N*-dimethyl-3-ammonio-1-propanesulfonate, 40 mM Tris and 0.2% (w/v) BioLyte 3/10 ampholyte with ultrasonic disruption. Three lysates were pooled as one biological replicate, and two biological replicates were used for further proteomic analysis. Proteins were precipitated using 20% (w/v) trichloroacetic acid /acetone solution at -20°C overnight, centrifuged at 20,000 g for 20 min at 4°C, and then washed twice using cold acetone. Finally, the precipitate was redissolved in the lysis buffer (7 M urea, 2 M thiourea, 2% SDS and 40 mM Tris).

After being reduced and alkylated, 100 μg protein from each sample was digested with Trypsin Gold (Promega, United States) in a 10 KDa ultrafiltration device (Millipore, United States), desalted using Strata X column (Phenomenex, United States), dried by a vacuum centrifuge and reconstituted in 0.5 M TEAB. Peptides were labeled using 8-plex iTRAQ reagent (Applied Biosystems, United States). Eight samples (two biological replicates for each sample) that were labeled with different iTRAQ tags (113, 114, 115, 116, 117, 118, 119, and 121) were pooled and dried using vacuum centrifugation.

### Peptide Fractionation and LC -MS/MS Analysis

The dried peptide samples were reconstituted with 2 mL buffer A (5% acetonitrile (ACN), pH 9.8) and fractionated with a 4.6 mm × 250 mm Gemini C18 column (Phenomenex, United States) using an LC-20AB HPLC pump system (Shimadzu, Japan). Peptides were eluted at a rate of 1 mL/min with a gradient of 5% buffer B (95% ACN, pH 9.8) for 10 min, 5% to 35% buffer B for 40 min, 35% to 95% buffer B for 1 min, maintained at buffer B for 3 min and then returned to 5%. The 20 fractions collected based on the elution profile were monitored at 214 nm wave length, and dried using vacuum centrifugation.

Each fraction was redissolved in buffer C (2% ACN, 0.1% formic acid (FA)), separated on an LC-20AD nano HPLC (Shimadzu, Japan), and followed by the analysis of tandem mass spectrometry (MS/MS) Q-Exactive (Thermo Fisher Scientific, San Jose, CA, United States) after nanoelectrospray ionization. Peptides were separated at a flow rate of 300 nL/min with an effective gradient of 5% buffer D (98% ACN, 0.1% FA) for 8 min, 8 to 35% buffer D for 35 min, 35 to 60% buffer D for 5 min, 60 to 80% buffer D for 2 min, and maintained at 80% buffer D for 5 min and then returned to 5%.

The MS scans were operated at a resolution of 70,000 and the MS/MS scans were at a resolution of 17,500. The 20 most abundant precursor ions above a threshold intensity of 10,000 with a 2+ to 7+ charge-state were selected for MS/MS using high-energy collision dissociation. Ion Fragments were detected in the Orbitrap. The dynamic exclusion duration was set as 15 s.

### Bioinformatics Analysis

Raw MS files were integrated and transformed using a Proteome Discoverer (Thermo Fisher Scientific, San Jose, CA, United States). The peptides and proteins were identified using the Mascot search engine (ver. 2.3.02; Matrix Science, London, United Kingdom). The main parameters were as follows: fragment mass tolerance of 0.05 Da, peptide mass tolerance of 20 ppm, PSM-level FDR within 0.01 and protein-level FDR within 0.01. NCBI reference sequences of *T. pseudonana*^1^ were used as the protein database for identification. Each protein identified had to contain at least one unique peptide and two unique spectra. The proteins were annotated by blasting against NCBInr, SwissProt/UniProt, COG, GO and the KEGG database.

Proteins with at least two unique peptides were considered for protein quantitation. DEPs were defined with the criteria of mean fold change >1.5 or <0.67 (compared to the control), and *P*-value < 0.05. The KEGG pathway enrichment analysis was conducted and evaluated by hypergeometric test.

### RNA Extraction and Quantitative PCR Analysis

Representative genes involved in key biological processes were selected for the quantitative PCR (qPCR) analysis to verify the corresponding protein expressions under the N-, P-, and Si deficient conditions. Three biologically replicates for each treatment were lysed in Trizol reagent (1 mL, Life Technologies) and extracted RNA following the instruction manual of the RNA isolation Kit (Cat No. 74104, Qiagen, Germany). Total purified RNA was treated with DNase and then immediately transcribed to cDNA using the Reverse Transcription Kit (Cat. No. 205311, Qiagen, Germany). Reverse transcription was carried out at 42°C for 25 min and inactivated at 95°C for 2 min. The cDNA samples were maintained at 4°C for immediate use.

Target cDNA sequences were obtained from NCBI database according to the accession numbers of identified proteins. Primers were designed using the online Primer3 Input (v0.4.0) (Supplementary Table S1). PCR products of cDNA were separated on agarose gels and sequenced to ensure the specificity and accuracy as described by Li et al. (2018).

qPCR reaction was conducted in an ABI 7500 Real-Time PCR System with mixture comprised 10 μL SYBR Premix, 2 μL of paired primer (0.4 μmol/L final concentration), 2 μL cDNA (total mass between 10 and 30 ng), 0.4 μL 50 × ROX Reference Dye and 6 μL sterilized H2O, all provided in the SuperReal PreMix Plus kit (Cat. No. 208054, Qiagen, Germany). The qPCR assay was initiated at 95°C for 10 min, followed by 40 cycles of 95°C for 15 s and 60°C for 1 min. Amplification curves and melt curves were monitored to test the integrity and stringency of the reactions. Relative gene expression was calculated as described by Li et al. (2018) and normalized using the internal control gene (actin transcript) (McGinn and Morel, 2008).

## Results

### Physiological Responses of *T. pseudonana* to Different Macronutrient Deficiencies

**FIGURE 1:**
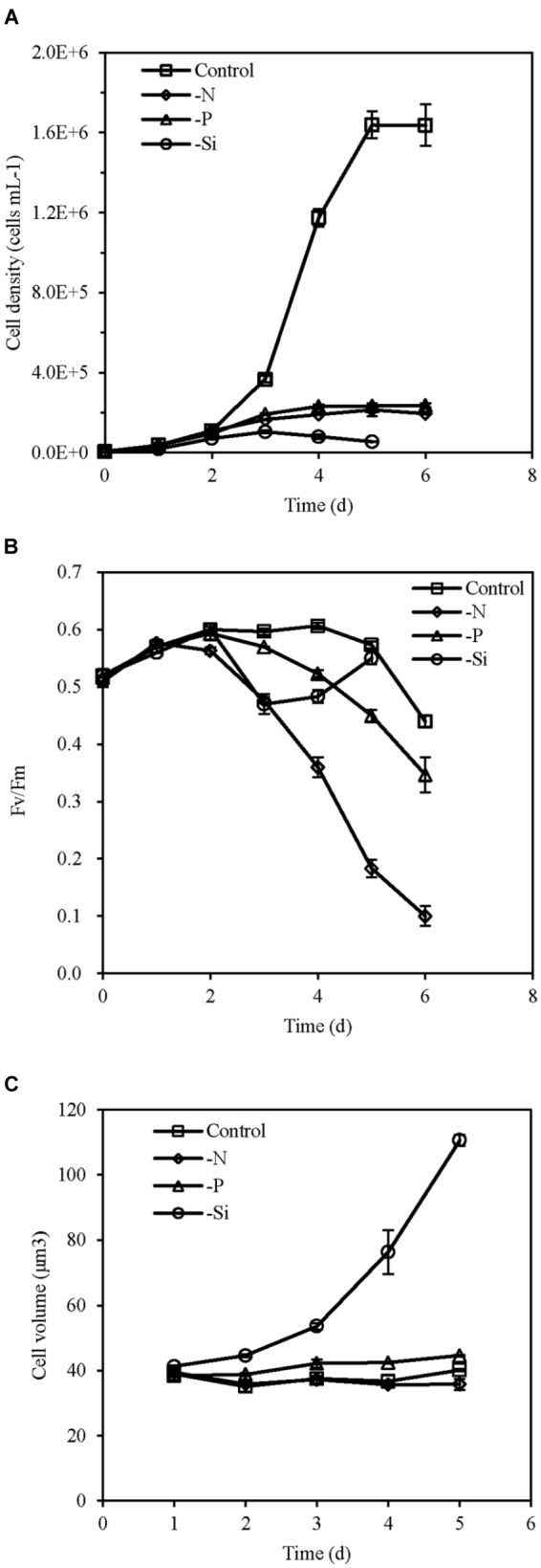
Cell density **(A)**, Fv/Fm **(B)**, and cell volume **(C)** of *T. pseudonana* grown under different macronutrient deficiencies (N deficiency, P deficiency, Si deficiency, and nutrient replete conditions). Error bars represent the standard deviations of the means generated from triplicates.

Cell density of the nutrient-replete culture increased rapidly and entered the exponential growth phase in day 2 and the stationary growth phase in day 5. However, cells of N-, P-, and Si-deficient cultures grew slowly and growth ceased in day 3 or 4 (Figure 1A). The Fv/Fm of all cultures increased in the first 2 days, and then was maintained at a relatively stable high level (about 0.6) in the nutrient-replete cells. However, it was markedly reduced in the N- and P-deficient cells from day 2 (Figure 1B). The Fv/Fm of the Si-deficient cells decreased in day 2 but increased from day 3 (Figure 1B). Cell volumes of the P- and Si-deficient cells became larger with the growing time compared with the nutrient-replete cells, especially the Si-deficient cells, while it altered insignificantly in the N-deficient cells (Figure 1C).

**FIGURE 2:**
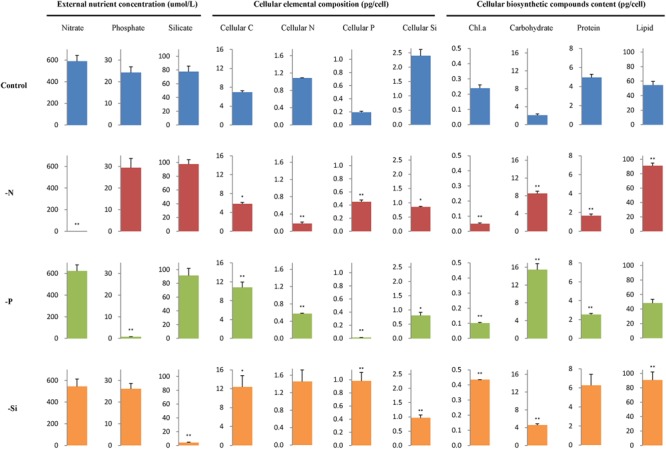
External nutrient concentration, cellular elemental composition, and biosynthetic compounds content per cell of *T. pseudonana* in different macronutrient deficient conditions. Error bars represent the standard deviations of the means generated from triplicates. ^∗∗^*P* < 0.01 and ^∗^*P* < 0.05 indicate significant correlation.

Concentrations of nutrients in the culture media, contents of cellular element and biosynthetic compounds are shown in Figure 2. Concentrations of nitrate, phosphate, and silicate were very low in each individual macronutrient deficient culture medium. Cellular C content per cell increased in the P- and Si-deficient cells compared with the nutrient-replete cells, but it decreased in the N-deficient cells. Cellular N content decreased but cellular P content increased in the N-deficient cells, while cellular N and P contents decreased in the P-deficient cells. However, cellular P content increased in the Si-deficient cells compared with the nutrient-replete cells. Cellular Si content decreased in all macronutrient-deficient cells.

In addition, contents of chlorophyll a and protein decreased in the N- and P-deficient cells but content of chlorophyll a increased in the Si-deficient cells. Lipid content increased in the N- and Si-deficient cells but altered insignificantly in the P-deficient cells. Carbohydrate content increased in all macronutrient deficient cells, especially in the P-deficient cells, with an approximately 6.3-fold increase.

Contents of cellular element and biosynthetic compounds were also normalized to mean cell volume (Supplementary Table S2). They presented the same variation patterns as their contents normalized to per cell. However, total lipid and cellular C contents per volume altered insignificantly in the Si-deficient cells.

### Proteomics Overview

In total, 84094 of the whole output of 352372 spectra were matched to 25001 peptides, resulting in the identification of 24620 unique peptides and 5482 proteins with at least one unique peptide. Among them, 3798 high-confidence proteins matched by two or more unique peptides were selected to perform protein quantification (Supplementary Table S3).

**FIGURE 3:**
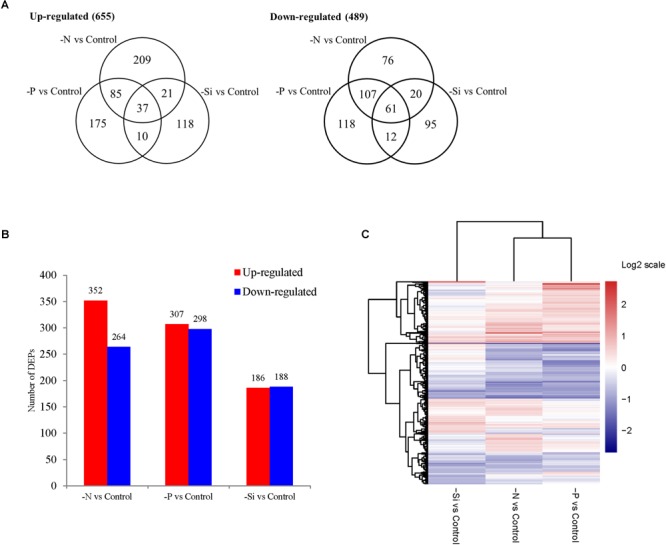
The Venn diagram **(A)**, the number **(B)**, and the hierarchical cluster **(C)** of DEPs in *T. pseudonana* in N-, P-, and Si- deficient cells relative to the nutrient-replete cells.

Of the total 3798 quantified proteins, 1111 were differentially expressed proteins (DEPs) among three macronutrient deficient cells compared with the nutrient-replete cells (Supplementary Table S4). In the N-deficient cells, 352 proteins were up-regulated and 264 were down-regulated, while 307 proteins were up-regulated and 298 were down-regulated in the P-deficient cells (Figure 3A). Among them, 122 up-regulated proteins and 168 down-regulated proteins were shared in both N- and P-deficient cells (Figure 3B). In the Si-deficient cells, 186 proteins were up-regulated and 188 were down-regulated. In both N- and Si-deficient cells, 58 up-regulated proteins and 81 down-regulated proteins were shared, while 47 up-regulated proteins and 73 down-regulated proteins were shared in both P- and Si-deficient cells. Among all DEPs, 37 up-regulated proteins and 61 down-regulated proteins were shared among three macronutrient deficient cells. The hierarchical clustering of all DEPs indicated that the N- and P-deficient cells shared more common DEPs compared with the Si-deficient cells (Figure 3C).

**FIGURE 4:**
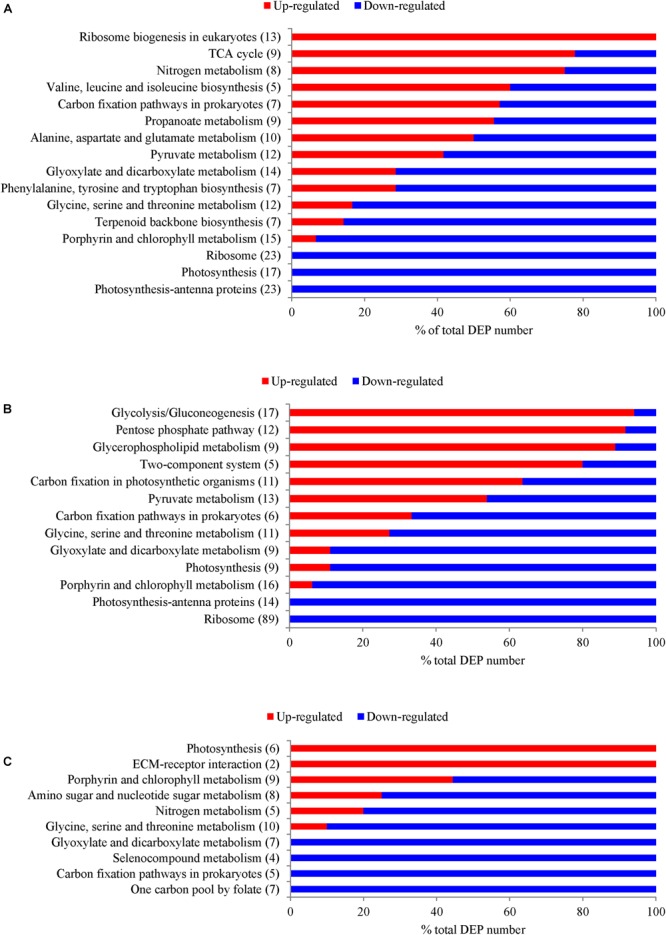
The number of DEPs in *T. pseudonana* from several crucial KEGG pathways that were significantly enriched with a *p*-value of less than 0.05 under different macronutrient deficiencies. **(A)** DEPs under N-deficiency; **(B)** DEPs under P-deficiency; **(C)** DEPs under Si-deficiency. Changes are denoted as the percentage of up-regulated (red) and down-regulated (blue) genes within each pathway.

Significantly enriched KEGG pathways with a *p*-value of less than 0.05 are shown in Figure 4 and Supplementary Table S5. Pathways, including “Ribosome,” “Photosynthesis,” “Photosynthesis antenna proteins” (“Light-harvesting chlorophyll protein complex (LHC)”), “Porphyrin and chlorophyll metabolism,” “Carbon fixation,” and “Glyosylate and dicarboxylate metabolism,” were down-regulated in both N- and P-deficient cells. “Ribosome biogenesis,” “TCA cycle,” and “Nitrogen metabolism” were highly expressed in the N-deficient cells. In addition, pathways involved in amino acid biosynthesis and metabolism were also enriched. “Glycolysis/Gluconeogenesis,” “Pentose phosphate pathway,” “Glycerophospholipid metabolism,” and “Two-component system” were up-regulated in the P-deficient cells. Pathways including “One carbon pool by folate,” “Carbon fixation,” “Selenocompound metabolism,” “Glyoxylate and dicarboxylate metabolism,” “Nitrogen metabolism,” and “Glycine, serine, and threonine metabolism” were down-regulated in the Si-deficient cells. However, “Photosynthesis” was up-regulated in these cells compared with the nutrient-replete cells.

### DEPs Involved in Nutrient Transport and Utilization

In the N-deficient cells, transporters, such as nitrate/nitrite transporters (NRT1 and NRT2), urea transporters, xanthine uracil permease (TN.NCS2) and amino acid transporter, were significantly up-regulated (Table 1). Among them, TN.NCS2 was 8.48-fold up-regulated and urea-proton symporter (DUR3) was 6.14-fold up-regulated. Meanwhile, the abundances of glutamate dehydrogenase, glutamate synthase and glutamine synthetase were increased. In addition, urease (URE), nitrilase, amidase, and purine degradation proteins were also highly expressed.

**Table 1:** The DEPs involved in the transport and utilization of nitrogen, phosphate, and silicon sources in *T. pseudonana* under different macronutrient stresses.

Protein name	Accession number	Number of unique peptide	Fold change
N deficiency			(-N vs. Control)
Transport			
Nitrate/nitrite transporter (NRT1)	XP_002288802	3	1.56
Nitrate/nitrite transporter (NRT2)	XP_002295904	3	2.13
3 Urea-proton symporter (DUR3)	XP_002292926	3	6.14
Urea transporter (SLC14A)	XP_002295777	2	1.8
Xanthine/uracil permease (TN.NCS2)	XP_002295239	2	8.48
Sodium-coupled neutral amino acid transporter	XP_002291100	4	1.81
Nitrogen metabolism			
Glutamine synthetase (GLNN)	XP_002295274	32	1.66
Glutamate synthase (NADPH/NADH) (GLT1)	XP_002293590	54	2.12
Glutamate synthase (NADPH/NADH) small chain (gltD)	XP_002291583	20	2.38
Glutamate dehydrogenase	XP_002289225	15	1.57
Aliphatic amidase	XP_002289996	8	1.73
Nitrilase (NIT2)	XP_002290043	4	1.63
Purine degradation			
AMP deaminase	XP_002289781	3	1.71
5-hydroxyisourate hydrolase	XP_002288652	6	1.61
Allantoicase	XP_002289615	7	1.54
Urease (URE)	XP_002296690	22	1.58
P deficiency			(-P vs. Control)
Transport			
Sodium phosphate co-transporter (SLC34A)	XP_002292964	6	6.5
Phosphate transport protein (PTP1)	XP_002287546	12	1.55
Phosphorus utilization			
Alkaline phosphatase (phoD)	XP_002294783	16	5.52
Alkaline phosphatase (phoA, phoB)	XP_002286339	10	7.02
Alkaline phosphatase (AP)	XP_002286092	21	6.52
5′-nucleotidase/UDP-sugar diphosphatase (ushA)	XP_002295546	11	7.36
5′-nucleotidase	XP_002295180	10	1.8
ADP-ribose pyrophosphatase (nudF)	XP_002287086	3	1.72
Cytokinin riboside 5′-monophosphate nucleosidase	XP_002291575	5	1.61
Phosphoserine phosphatase	XP_002287672	5	1.53
Glycerophosphoryl diester phosphodiesterase (glpQ)	XP_002292125	13	7.01
Phospholipase D1/2 (PLD1_2)	XP_002288407	18	1.97
Phosphatidylinositol phosphodiesterase	XP_002292372	2	1.57
Phosphatidylserine decarboxylase	XP_002295759	8	2.94
Polyphosphate allocation			
Vacuolar transporter chaperone 4 (VTC4)	XP_002295322	27	3.63
N containing lipids synthesis			
Betaine aldehyde dehydrogenase	XP_002295797	8	1.58
Si deficiency			(-Si vs. Control)
Transport			
Silicic acid transporter (SIT1)	XP_002290700	3	8.62
Silicic acid transporter (SIT2)	XP_002295920	5	6.17

Two phosphate transporters were more abundant in the P-deficient cells, especially sodium phosphate co-transporter (SLC34A) was 6.5-fold up-regulated (Table 1). Vacuolar transporter chaperone 4 (VTC4), related to cellular P allocation, was more abundant. Three alkaline phosphatases were significantly up-regulated by 5.52, 7.02, and 6.52-fold. The abundances of a number of enzymes capable of cleaving nucleoside diphosphate and phosphatidylserine were also increased in the P-deficient cells; in particular, 5′-nucleotidase/UDP-sugar diphosphatase (ushA) was 7.36-fold up-regulated. In addition, several enzymes involved in degradation of phospholipids were highly expressed. Among them, glycerophosphoryl diester phosphodiesterase (glpQ) was 7.01-fold up-regulated.

In the Si-deficient cells, two silicic acid transporters (SITs), SIT1 and SIT2 specifically recognizing and transporting silicic acid across lipid bilayer membranes, were significantly up-regulated by 8.62 and 6.17-fold (Table 1).

### DEPs Involved in Photosynthesis and Chloroplastic F-type ATPase

**FIGURE 5:**
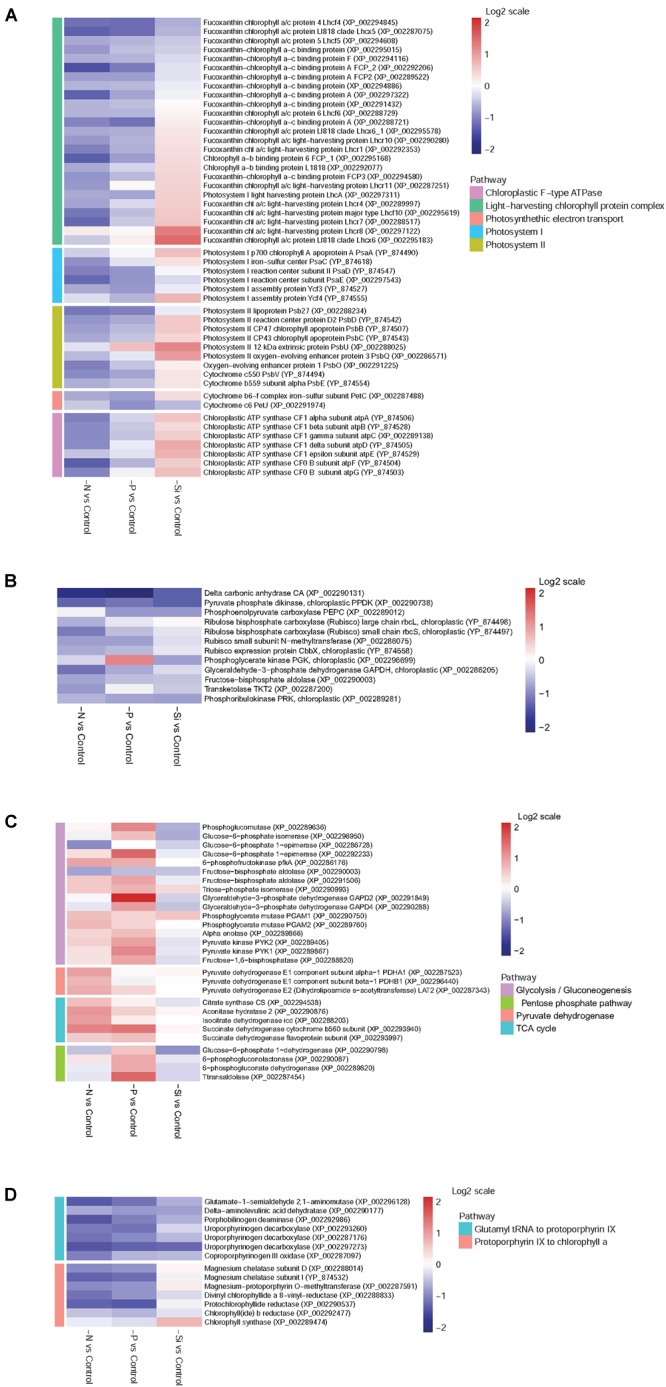
Heat maps of DEPs in *T. pseudonana* from key pathways for different expressions in the N-, P-, and -Si deficient cells relative to the nutrient-replete cells. **(A)** Photoreaction and chloroplastic ATPase; **(B)** Carbon fixation; **(C)** Carbohydrate metabolism; **(D)** Chlorophyll biosynthesis. Each nutrient condition corresponds to a single column and each protein to a single row. The color chart indicates fold change of protein expression using a base 2-logarithmic scale. The color scale ranges from saturated firebrick for up-regulated proteins to saturated navy for down-regulated proteins; white indicates no significant change.

Forty-nine DEPs involved in photosynthesis and chloroplastic F-type ATPase were classified into five groups: “LHC,” “Photosystem I (PS I),” “PS II,” “Photosynthetic electron transport,” and “Chloroplastic F-type ATPase.” Overall, most of the proteins associated with photosynthesis and light-harvesting were down-regulated in both N-and P-deplete cells (Figure 5A). However, more proteins involved in photosynthesis and light-harvesting were up-regulated in the Si-deficient cells. Among them, LHC proteins, especially Lhcx6, Lhcr8, and Lhcr7, and almost all subunits of chloroplastic F-type ATPase were significantly increased in abundance.

### DEPs Involved in Carbon Fixation and Carbohydrate Metabolism

Delta carbon anhydrase (CA) was markedly down-regulated among all macronutrient deficient cells, especially in the P-deficient cells (Figure 5B). Two subunits of ribulose bisphosphate carboxylase (Rubisco), rbcL and rbcS, were significantly down-regulated in the N-deficient cells, while they altered insignificantly in the P- and Si-deficient cells. Pyruvate phosphate dikinase (PPDK) and phosphoenolpyruvate carboxylase (PEPC), two key enzymes of the C4 cycle, were down-regulated in both P- and Si-deficient cells, while pyruvate phosphate dikinase was down-regulated in the N-deficient cells. Most DEPs related to the Calvin cycle were down-regulated among all macronutrient deficient cells (Figure 5B).

Many enzymes related to glycolysis were significantly up-regulated in the P-deficient cells, including 6-phosphofructokinase (pfkA) and pyruvate kinase (PYK1 and PYK2). Glyceraldehyde 3-phosphate dehydrogenase GAPD2, catalyzing the sixth step of glycolysis and forming NADH, was increased 4.27-fold in abundance (Figure 5C). In the N-deficient cells, expression of pfkA was increased. However, most enzymes associated with glycolysis and gluconeogenesis altered insignificantly in the Si-deficient cells. Three components of pyruvate dehydrogenase (PDHA1, PDHB1, and LAT2) involved in the pyruvate metabolism pathway were up-regulated in the N-deficient cells, while they altered insignificantly in both P- and Si-deficient cells. In addition, D-lactate dehydrogenase was markedly increased in abundance in the P-deficient cells. Proteins involved in the TCA cycle were up-regulated in both N- and P-deficient cells, especially in the N-deficient cells, while they altered insignificantly in the Si-deficient cells. In addition, four proteins involved in the pentose phosphate pathway (PPP) were up-regulated in the P-deficient cells, but they were down-regulated in both N-and Si-deficient cells.

### DEPs Involved in Chlorophyll Biosynthesis

Most enzymes involved in chlorophyll biosynthesis were down-regulated in both N- and P-deficient cells (Figure 5D). Despite several enzymes involved in chlorophyll synthesis from glutamyl tRNA to protoporphyrin IX were also down-regulated in the Si-deficient cells, enzymes from protoporphyrin IX to chlorophyll a altered insignificantly and a chlorophyll synthase (chlG) was exclusively up-regulated.

### Ribosomal Proteins

**FIGURE 6:**
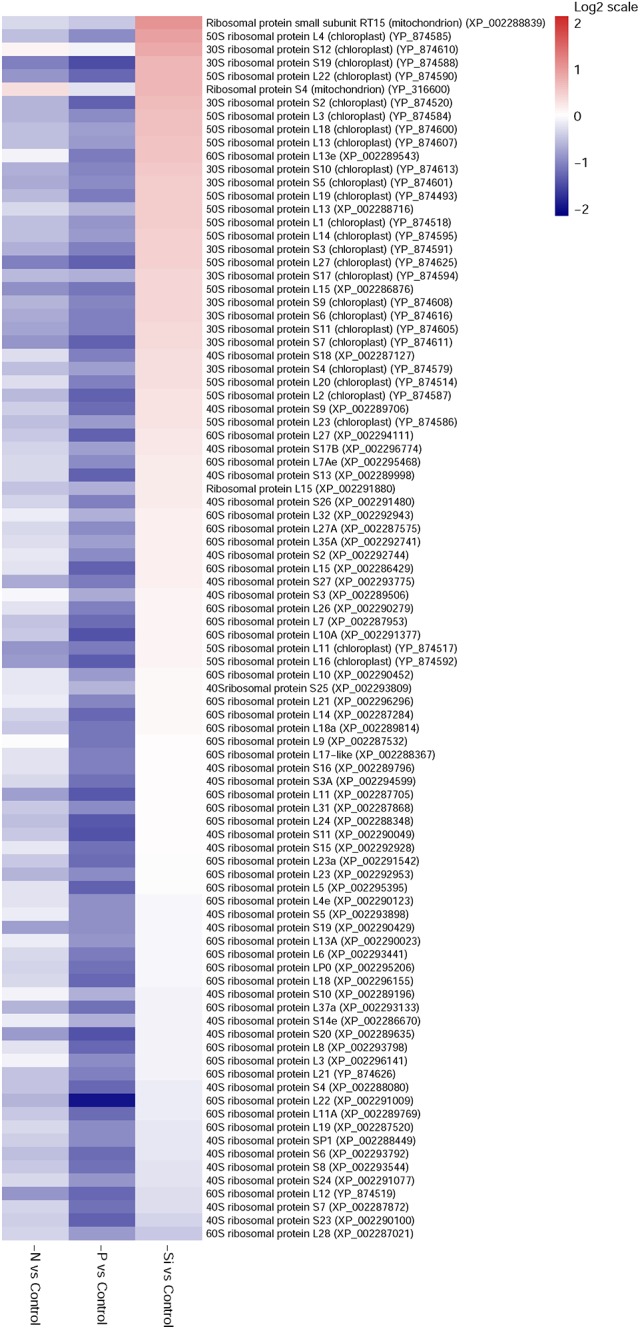
Heat maps of differentially expressed ribosomal proteins in *T. pseudonana* for different expressions in the N-, P-, and -Si deficient cells relative to the nutrient-replete cells. Each nutrient condition corresponds to a single column and each protein to a single row. The color chart indicates fold change of protein expression using a base 2-logarithmic scale. The color scale ranges from saturated firebrick for up-regulated proteins to saturated navy for down-regulated proteins; white indicates no significant change.

Eight-nine ribosomal proteins in the P-deficient cells, and 23 in the N-deficient cells, were identified; and all of them were down-regulated (Figure 6). However, 11 ribosomal proteins were up-regulated in the Si-deficient cells, and most of them were located in the chloroplast.

### Validation of DEPs Using qPCR

**FIGURE 7:**
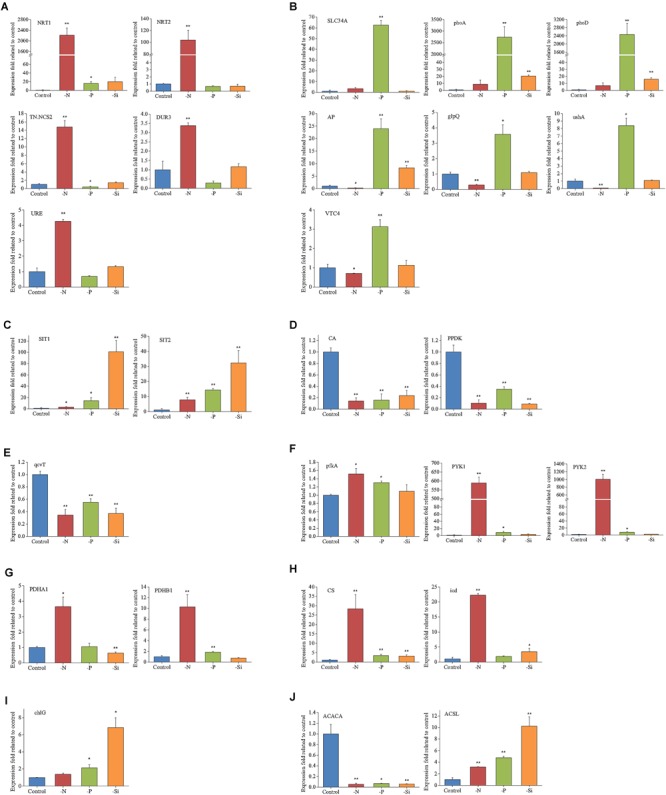
Relative transcripts of selected genes from key biological processes in *T. pseudonana* in the N-, P-, and -Si deficient cells relative to the nutrient-replete cells. **(A)** N transport and utilization; **(B)** P transport and utilization; **(C)** Si transport; **(D)** Carbon fixation; **(E)** Photorespiration; **(F)** Glycolysis; **(G)** Pyruvate dehydrogenase; **(H)** TCA cycle; **(I)** Chlorophyll synthase; **(J)** Lipid synthesis. Error bars represent the standard deviations of the values generated from three biological replicates. ^∗∗^*P* < 0.01 and ^∗^*P* < 0.05 indicate significant correlation.

Expressions of 27 representative genes under different nutrient conditions are shown in Figure 7. Expressions of *NRT1, NRT2, TN.NCS2, DUR3*, and *URE* were increased remarkably in the N-deficient cells, while *SLC34A*, three alkaline phosphatases (*phoD, phoA*, and *AP*), *ushA, glpQ*, and *VTC4* were increased significantly in the P-deficient cells. Two silicic acid transporter genes, *SIT1* and *SIT2*, were also up-regulated in the Si-deficient cells. *PPDK* and *CA*, two key enzymes involved in C4 cycle and carbon fixation, were significantly down-regulated in the N-, P-, and Si-deficient cells. Transcript expression of glycine decarboxylase T protein (*qcvT*), which is required for photorespiration, was obviously decreased in the N-, P-, and Si-deficient cells. Transcript abundances of p*fkA, PYK1* and *PYK2*, rate-limiting enzymes in glycolysis, were significantly increased in the N- and P-deficient cells, especially in the N-deficient cells. However, protein abundances of PYK1 and PYK2 altered insignificantly in the N-deficient cells (Figure 5C). Two key enzymes of TCA cycle, citrate synthase (*CS*) and isocitrate dehydrogenase (*icd*), and two subunits of pyruvate dehydrogenase (*PDHA1* and *PDHB1*) were up-regulated greatly in the N-deficient cells. An acetyl-CoA carboxylase (*ACACA*) restricting the first stage of fatty acid synthesis, was down-regulated, while a long-chain acyl-CoA synthetase (*ACSL*) was significantly up-regulated in the N-, P- and Si-deficient cells. Transcript expression of *chlG*, the last step of chlorophyll biosynthesis process, was specifically up-regulated in the Si-deficient cells. It should be pointed out that the change trends of genes were not completely consistent with proteins, which might be caused by the post-transcriptional regulation or by the difference in temporal expression of genes and proteins (Dyhrman et al., 2012).

## Discussion

### Specific Nutrient Transport and Utilization Proteins Under a Particular Macronutrient Deficiency

The N- and P-deficient cells not only up-regulated the corresponding inorganic nutrient transporters, but also increased utilization of organic nutrients. In the N-deficient cells, two high-affinity urea transporter-type transporters, DUR3 and SLC14A (Solomon et al., 2010), and URE were significantly up-regulated (Table 1 and Figure 7), indicating that urea is an important alternative *N* source for diatoms in the ambient N-deficient condition. Notably, TN.NCS2 presented the highest fold increase in the protein level (Table 1) and high expression in the transcriptional level (Figure 7). Expression of TN.NCS2 is influenced by N availability and forms (Wurch et al., 2014), and it is detected only in the cells grown on nitrate when cell growth is limited by nitrate (Wurch et al., 2011). In addition, 5-hydroxyisourate hydrolase and allantoicase, two enzymes involved in purine degradation, were up-regulated (Table 1), suggesting that purine was utilized as the secondary *N* source under the N-deficient condition. Moreover, allantoicase converts allantoate to ureidoglycolate and urea, where urea enters the assimilation pathway via urease (Vogels and Van Der Drift, 1976). It is not surprising that various aminohydrolases, including aliphatic amidase, nitrilase, and AMP deaminase, were up-regulated to produce more ammonia. Aliphatic amidase and nitrilase show significant homology with each other and both play critical roles in N utilization (Novo et al., 1995; Howden and Preston, 2009). AMP deaminase catalyzes the deamination of AMP to IMP and releases an ammonia molecule. Since AMP deaminase is indispensable in the purine nucleotide cycle (Moffatt and Ashihara, 2002), we speculated that this enzyme played a key role in purine degradation.

Any intracellular or extracellular N must first be converted to ammonium before assimilation into amino acids and other nitrogenous compounds. To maximize the assimilation of N, key enzymes involved in the N assimilation pathway, such as glutamine synthetase, glutamate dehydrogenase, and glutamate synthase, were up-regulated in the N-deficient cells (Table 1). Glutamate plays the central role in intracellular N flow, serving as both N donor and N acceptor (Miflin and Habash, 2002). Overall, *T. pseudonana* responded to ambient N deficiency through increase of N transport, assimilation and utilization of organic N nutrients, which was consistent with the transcriptomic and metabolic results reported in *T. pseudonana* and *P. tricornutum* (Bender et al., 2014; Alipanah et al., 2015).

A previous transcriptomic and proteomic study highlights that *T. pseudonana* has evolved multiple response mechanisms to ambient P-deficiency, for example, changing cellular P allocation, utilizing DOP and non-P-containing lipids alternately, in addition to inorganic P transport (Dyhrman et al., 2012). Polyphosphate can serve as a storage compound of intracellular inorganic phosphate. VTC4 is a part of the vacuolar transporter chaperone complex that interacts with the vacuole membrane and produces polyphosphate in yeast (Ogawa et al., 2000; Hothorn et al., 2009). This protein was significantly up-regulated in the P-deficient cells in both mRNA and protein levels (Table 1 and Figure 7), indicating re-allocation of cellular P. In addition, alkaline phosphatases, 5′-nucleotidases, phospholipases, and phosphodiesterases were also highly expressed in both mRNA and protein levels (Table 1 and Figure 7), suggesting that the P-deficient cells initiated another strategy to acquire P from organic compounds through increasing the expressions of various phosphatases. This result was consistent with a recent transcriptomic and proteomic study of *T. pseudonana* as well as studies on the enzymatic activities (Dyhrman and Palenik, 2003; Yamaguchi et al., 2005; Dyhrman et al., 2012). Phytoplankton can use non-P lipids, such as sulfolipids (sulfur containing) and betaine lipids (N containing), to replace P containing lipids in response to ambient P-deficiency (Van Mooy et al., 2009). In our study, a betaine aldehyde dehydrogenase related to betaine lipid biosynthesis (Sakamoto and Murata, 2000) was up-regulated in the P-deficient cells (Table 1). However, the proteins involved in sulfolipid biosynthesis altered insignificantly, which differed from a previous transcriptomic and proteomic study that two genes related to sulfolipid biosynthesis are up-regulated in the P-deficient *T. pseudonana* (Dyhrman et al., 2012).

SITs are responsible for silicic acid transport through the plasma membrane for the formation of the diatom frustule (Hildebrand et al., 1997, 1998). Three SIT encoding genes are predicted in the *T. pseudonana* genome, and SIT1 and SIT2 were significantly up-regulated in the Si-deficient cells in our study (Table 1 and Figure 7), which was consistent with the result of previous transcriptomic and proteomic studies of *T. pseudonana* (Shrestha et al., 2012; Du et al., 2014). Ambient Si deficiency could facilitate transport of Si to meet the demand of cells for Si.

### Common Responses of *T. pseudonana* to N- and P-Deficiencies

**FIGURE 8:**
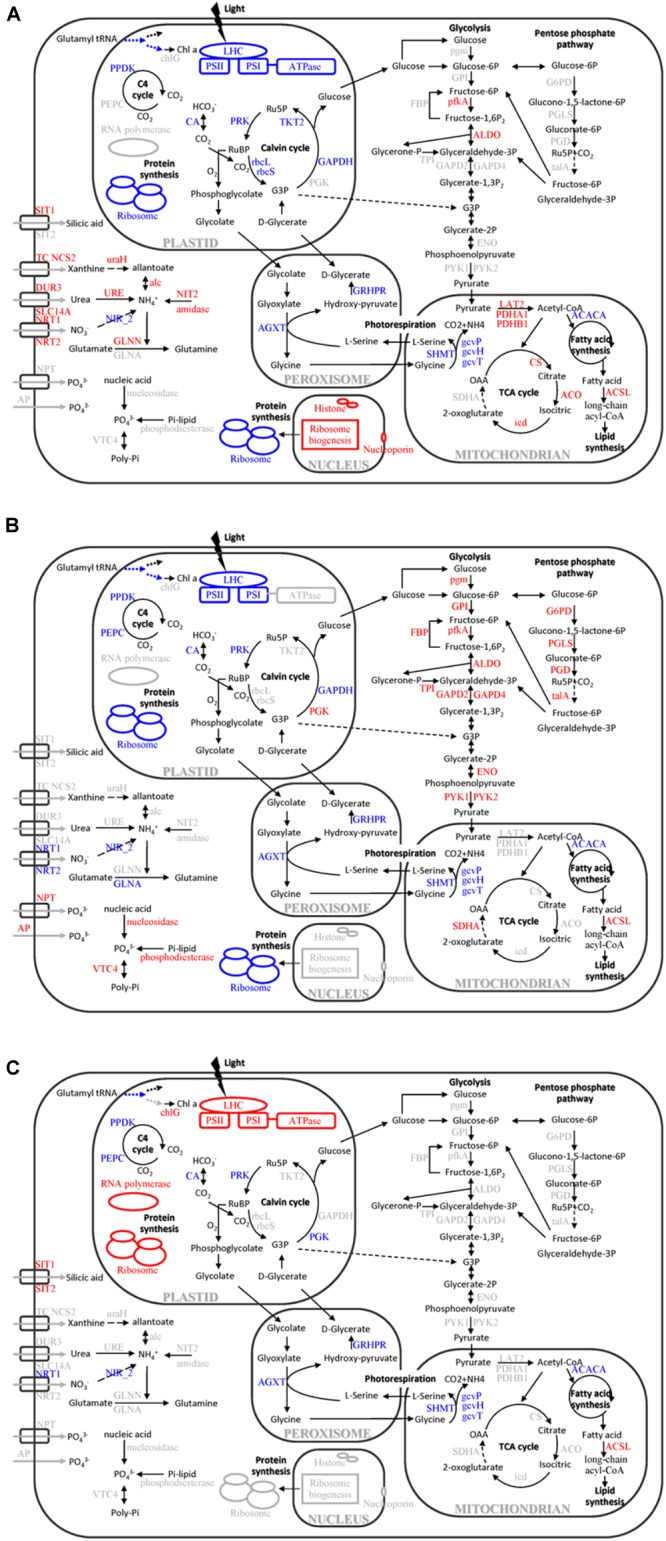
Cellular pathways and processes affected by different macronutrient deficiencies in *T. pseudonana*. **(A)** N-deficiency; **(B)** P-deficiency; **(C)** Si-deficiency. Red, blue, and black texts indicate up-regulation, down-regulation and no change of pathways or proteins. OAA, oxaloacetate; RuBP, ribulose-1,5-bisphosphate; Ru5P, ribulose-5P; G3P, glycerate-3P; Chl a, chlorophyll a; chlG, chlorophyll synthase; LHC, light-harvesting chlorophyll protein complex; PPDK, pyruvate phosphate dikinase; PEPC, phosphoenolpyruvate carboxylase; CA, carbonic anhydrase; rbcL, Rubisco large chain; rbcS, Rubisco small chain; PRK, phosphoribulokinase; TKT2, fructose-bisphosphatealdolase; PGK, phosphoglycerate kinase; GAPDH, glyceraldehyde-3-phosphate dehydrogenase; SIT, silicic acid transporter; TC.NCS2, xanthine/uracil permease; NRT, nitrate/nitrite transporter; DUR3, urea-proton symporter; SLC14A, urea transporter; NPT, sodium phosphate co-transporter; AP, alkaline phosphatase; uraH, 5-hydroxyisourate hydrolase; URE, urease; alc, allantoicase; NIT2, nitrilase; NIR_2, Ferredoxin-nitrite reductase; GLN, glutamine synthetase; VTC4, vacuolar transporter chaperone 4; SHMT, glycine/serine hydroxymethyltransferase; AGXT, alanine-glyoxylate transaminase; gcvP, glycine decarboxylase P protein; gcvH, glycine decarboxylase H protein; gcvT, glycine decarboxylase T protein; GRHPR, glycerate dehydrogenase/hydroxypyruvatereductase; PDHA1, pyruvate dehydrogenase E1 component subunit alpha-1; PDHB1, pyruvate dehydrogenase E1 component subunit beta-1; LAT2, pyruvate dehydrogenase E2 (dihydrolipoamide s-acetyltransferase); CS, citrate synthase; ACO, aconitasehydratase 2; icd, isocitrate dehydrogenase; SDHA, succinate dehydrogenase flavoprotein subunit; ACACA, acetyl-CoA carboxylase; ACSL, long-chain acyl-CoA synthetases; G6PD, glucose-6-phosphate 1-dehydrogenase; PGLS, 6-phosphogluconolactonase; PGD, 6-phosphogluconate dehydrogenase; talA, ttransaldolase; pgm, phosphoglucomutase; GPI, glucose-6-phosphate isomerase; FBP, fructose-1,6-bisphosphatase; pfkA, 6-phosphofructokinase; ALDO, fructose-bisphosphatealdolase; TPI, triose-phosphate isomerase; GAPD, glyceraldehyde-3-phosphate dehydrogenase; ENO, alpha enolase; and PYK, pyruvate kinase.

Nitrogen and P participate in different physiological processes, and both of them are basic elements for protein synthesis. A previous study indicates that N or P starvation can decrease the synthesis rate of proteins, increase metabolic imbalance and oxidative stress, and negatively impact photosynthesis and carbon fixation (Bucciarelli and Sunda, 2003). In our study, proteins involved in chlorophyll biosynthesis, LHC, photosynthetic apparatus and the Calvin cycle were down-regulated in both N- and P-deficient cells (Figures 5, 8). Physiological results also showed the decrease of chlorophyll a and cellular N content in both N- and P-deficient cells (Figure 2). Previous transcriptomic studies of *P. tricornutum* also find that the majority of genes involved in photosynthesis and chlorophyll biosynthesis are depressed under N or P deficiency (Alipanah et al., 2015; Cruz de Carvalho et al., 2016). Generally, chlorophyll a and LHC proteins within the photosynthetic apparatus, and the enzymatic elimination of reactive oxygen species are all dependent on proteins that are rich in N. Expectedly, more proteins assigned to fucoxanthin chlorophyll a/c proteins and PS I and PS II were down-regulated in the N-deficient cells compared with the P-deficient cells. These results were consistent with the findings in the diatoms *Cyclotella cryptic* (Brakemann et al., 2006) and *P. tricornutum* (Yang et al., 2014), suggesting that N-deficiency damaged light reaction efficiency worse compared with P-deficiency.

Marine diatoms are responsible for up to 20% of global CO_2_ fixation (Nelson et al., 1995). Their photosynthetic efficiency is enhanced by CO_2_ concentration around Rubisco, but the mechanism is unclear. Despite the fact that the Rubisco involved in the first major step of carbon fixation was significantly down-regulated only in the N-deficient cells, CA, which is involved in CO_2_ concentrating mechanisms (CCMs) and delivering CO_2_ to the active site of Rubisco, and several proteins involved in the Calvin cycle, were down-regulated in both N- and P-deficient cells (Figures 5B, 7, 8), suggesting that ambient N- or P-deficiency suppressed C3 photosynthesis. Reduced carbon fixation could be caused by down-regulation of chlorophyll biosynthesis and the proteins involved in photosynthetic apparatus in the N-deficient cells, or limited energy supply due to the lack of NADPH and ATP in the P-deficient cells. In addition, *T. pseudonana* possesses the enzymatic apparatus to operate C4 photosynthesis (Reinfelder et al., 2000, 2004; Armbrust et al., 2004), including phosphoenolpyruvate carboxykinase, phosphoenolpyruvate carboxylase (PEPC) and pyruvate orthophosphate dikinase (PPDK). In our study, PPDK was down-regulated in both N- and P-deficient cells while PEPC was down-regulated only in the P-deficient cells (Figures 5B, 7, 8), indicating the suppression of C4 photosynthesis. The transcript abundances of PPDK and CA were also markedly decreased, which further demonstrated the changes in protein expression (Figure 7). Down-regulations of several genes related to carbon-concentrating mechanism are observed in the N-deficient cells in a transcriptome study (Alipanah et al., 2015). However, enzymes involved in carbon fixation do not show a specific transcript level trend in response to P deficiency in *T. pseudonana* and *P. tricornutum* (Dyhrman et al., 2012; Cruz de Carvalho et al., 2016).

Nitrogen or P deficiency induced the activation of central carbon metabolism in spite of its respective distinct preference. Glycolysis and the TCA cycle were up-regulated in N-deficient cells (Figures 5C, 8A). Increased expression of the pyruvate dehydrogenase complex in the N-deficient cells was also observed, which would direct carbon away from an intracellular carbohydrate store to the TCA cycle. Several key enzymes, i.e., pfkA, PYK1, PYK2, PDHA1, PDHB1, CS, and icd were up-regulated in mRNA level, despite PYK1 and PYK2 altered insignificantly in protein levels in the N-deficient cells (Figure 7). The TCA cycle in *T. pseudonana* is up-regulated in response to high levels of protein and amino acid degradation, which generates TCA cycle intermediates and provides precursors for N re-assimilation (Hockin et al., 2012; Bender et al., 2014). Up-regulations of glycolysis, TCA cycle, and pyruvate metabolism in proteomic level were consistent with their variation in transcriptional level in responses to N-deficiency in diatoms (Bender et al., 2014; Alipanah et al., 2015). However, in the P-deficient cells, only succinate dehydrogenase (SDH) was up-regulated. SDH catalyzes succinate-fumarate coupling and directly links the oxidation of TCA cycle intermediates to the reduction of the plastoquinone pool in the photosynthetic electron chain. In *Prochlorococcus* MED4, the SDH gene is lost so as to minimize its dependence on intracellular phosphate (Casey et al., 2016). Proteins involved in glycolysis were more significantly up-regulated in the P-deficient cells compared with the N-deficient cells, and proteins involved in the PPP exhibited a distinct up-regulation in the P-deficient cells (Figures 5C, 8B). Variations of glycolytic enzyme activities as a function of P-deficiency are observed in higher plants and green algae (Duff et al., 1989; Theodorou et al., 1991). Modulation of glycolysis related enzymes allows a P deficiency induced glycolytic bypass mechanism in diatoms (Dyhrman et al., 2012). The PPP is a metabolic pathway parallel to glycolysis, which generates NADPH and pentoses (5-carbon sugars) as well as ribose 5-phosphate, which is a by-product of nucleic acid degradation. These results suggested that P-deficiency activated processes involved in phosphate-reaction participation and the capacity of NADPH production, aiming to promote P utilization under P stress and energy supply under reduction of ATP.

Similar to previous studies of microarray, transcriptome and proteome in diatom and other species (Tetu et al., 2009; Gilbert and Fagan, 2011; Dyhrman et al., 2012; Bender et al., 2014; Alipanah et al., 2015), 23 ribosomal proteins in the N-deficient cells and 89 in the P-deficient cells were down-regulated. The sequences of ribosomal proteins are relatively N-rich (Acquisti et al., 2009). In addition, two nitrate transporters, a nitrite reductase and a glutamine synthetase GLNA were also down-regulated in the P-deficient cells (Figure 8 and Supplementary Table S4). N deficiency hampers protein synthesis and the ribosome may additionally act as a cellular “store” of N, which can be broken down when the cells suffer N shortage (Acquisti et al., 2009), while down-regulation of nitrate transport, N metabolism and ribosomal proteins under P deficiency was caused by a general reduction of cellular metabolic rate and energy supply limitation. Moreover, N derived from nucleotide degradation in the P- deficient cells (Table 1) could be utilized as the N source. These variations were also demonstrated by the decrease of protein content and cellular N in both N- and P- deficient cells (Figure 2).

However, N deficiency promoted P uptake (Figure 2). A group of proteins involved in ribosome biogenesis showed a specific increase in abundance under N-deficiency (Figure 4B), which could be considered as a negative feedback by the reduction of ribosomal proteins and a rapid recovery mechanism after N re-supplement. Thus, more P was required to be utilized as a component of RNA for ribosome biogenesis and ATP for protein synthesis. Another group of proteins localized in cell nucleus, including histone H1, H4 and nucleoporin SEH1 (Figure 8 and Supplementary Table S4), were up-regulated only in the N-deficient cells. The variation of these chromatin-associated proteins indicated the activation of intranuclear processes.

### Specific Response of *T. pseudonana* to Si-Deficiency

Differing from N and P, Si is the main component of the diatom frustule for cell protection, and Si deficiency resulted in a different protein expression compared with N or P deficiency. Three LHC proteins, Lhcx6, Lhcr8, and Lhcr7, were significantly up-regulated in the Si-deficient cells, but down-regulated in the N- and P-deficient cells (Figures 5A, 8). The result was similar to a recent proteomic study under high light stress (Dong et al., 2016), and Lhcx6 was observed to play a direct role in excess energy dissipation during high light stress (Zhu and Green, 2010). Smith et al. (2016) also find that the majority of genes annotated as LHC proteins including Lhcx6 are much more up-regulated in the microarray result than in the RNA-Seq result under Si starvation. In addition, the Si-deficient cells became much larger but contained less Si in the cell wall (Figure 2). Thus, we can assume that Si-deficiency might lead to the thinning of the frustule which increased light transmittance into the diatom cells, resulting in the excess of light for the cells. Thus, up-regulation of these proteins was a photoprotection to dissipate the excess light energy due to the abnormal Si-deficient cell walls of *T. pseudonana*.

ATP synthase plays a central role to convert the transthylakoid proton gradient to ATP. Activation of chloroplastic ATP synthase will decrease the proton gradient across the thylakoid membrane and enhance energy transduction between PS II and PS I, which results in a protective role during light stress (Braun et al., 1991). Increase of chloroplastic ATP synthase activity and accumulation of its subunits, such as the CF1 alpha and beta subunit, in response to high light stress are observed in previous studies (Chow et al., 1990; Burkey and Mathis, 1998; Jiao et al., 2004), indicating a positive response to alleviate the over protonation of thylakoid lumen and a prevention from photooxidative damage of the photosynthetic apparatus during high light stress. In addition, chloroplastic ATP synthase plays an important role in modulating non-photochemical quenching for photoprotection (Kanazawa and Kramer, 2002; Avenson et al., 2004). In our study, expressions of chloroplast ATP synthase, especially CF1 alpha, delta, epsilon subunits and CF0 B’ subunit, were significantly increased in the Si-deficient cells (Figures 5A, 8C and Supplementary Table S4). These results suggested that the peculiarly increasing expression of chloroplast ATP synthase might serve as an alternative photoprotection caused by Si-deficiency in *T. pseudonana*.

Up-regulations of many other chloroplast-encoded proteins were also observed only in the Si-deficient cells, and these proteins were involved in chloroplastic RNA transcription and protein biosynthesis. Differing from the N- and P-deficient cells, many ribosomal proteins were up-regulated in the Si-deficient cells, and most of them were located in the chloroplast of *T. pseudonana* (Figures 6, 8C). The increase of protein content was insignificant in the Si-deficient cells (Figure 2), which could be caused by the adjustment of protein synthesis among organelles. In addition, a chloroplastic DNA replication helicase and four plastid-encoded RNA polymerase subunits (alpha, beta, beta’ and beta” subunits) were also up-regulated (Figure 8 and Supplementary Table S4), consistent with the increase in cellular P content (Figure 2). These results indicated that chloroplast-related processes were enhanced in response to Si deficiency. A recent study shows that Si starvation results in larger chloroplasts and chloroplast replication, and the featured response at the transcriptional level is to regulate and coordinate cell cycle progression and chloroplast replication, rather than reduce Si availability and stress (Smith et al., 2016). The up-regulated processes in the chloroplast at protein level, including plastid DNA replication, transcription and protein biosynthesis, could be considered as the preparation for chloroplast division. Cell volume of the Si-deficient cells was much larger than that of the N- or P-deficient cells and the Fv/Fm was also increased after day 3 (Figures 1B,C), which might be caused by chloroplast replication in the cells. The increase of the Fv/Fm also could be explained by the increases of chloroplast located protein, chloroplast transcription and protein synthesis. This recovery phenotype could be considered as a delayed response to Si-deficiency, which gradually changed with chloroplast replication and cell volume. Moreover, chlorophyll a and cellular N content increased in the Si-deficient cells, contrary to the N- and P-deficient cells (Figure 2). Our proteomic results also showed that chloroplastic chlG was up-regulated only in the Si-deficient cells, in spite of the down-regulation of several enzymes related to chlorophyll *de novo* synthesis from glutamyl tRNA to protoporphyrin IX (Figure 5D). The up-regulation of chlG was also verified in mRNA level (Figure 7). Increasing the content of light-harvesting and photoprotective pigments are also documented under Si starvation which is regarded as the results of larger chloroplasts and chloroplast division (Holmes, 1966; Traller and Hildebrand, 2013; Smith et al., 2016).

### Common Responses of *T. pseudonana* to Different Macronutrient Deficiencies

Proteins involved in carbon fixation, including phosphoribulokinase, PPDK and CA were down-regulated in the N-, P-, and Si-deficient cells (Figures 5B, 7, 8), suggesting that all three macronutrient deficiencies suppressed the carbonic-anhydrase–dependent C4-like carbon concentrating pathway and impaired the Calvin cycle. Interestingly, a group of proteins involved in photorespiration were down-regulated in the N-, P-, and Si-deficient cells, including three component enzymes of the mitochondrial glycine decarboxylase (P-, H-, T-proteins), glycine/serine hydroxymethyltransferase, glycerate dehydrogenase/ hydroxypyruvate reductase and alanine-glyoxylate transaminase (Figure 8 and Supplementary Table S4). Among them, the up-regulation of gcvT was verified in mRNA level (Figure 7). Down-regulation of the energy-consuming processes of CCMs and photorespiration under a steady-state elevated CO_2_ concentration and nitrate limitation is also reported, which results in the metabolic re-arrangement of *T. pseudonana* (Hennon et al., 2015). These results indicated that *T. pseudonana* decreased energy-consuming photorespiration to survive in the poor nutrient condition in spite of its ability to reduce the free radicals. Moreover, despite carbon fixation being down-regulated, the cellular C content per cell increased in both P- and Si-deficient cells (Figure 2). We presume that this might be caused by the greater reduction of photorespiration. However, cellular C content decreased in the N-deficient cells, which might relate to the more down-regulated carbon fixation or carbon consumption by the TCA cycle.

Among the common DEPs, a ACSL was up-regulated in both protein and mRNA levels in the N-, P-, and Si-deficient cells (Figure 8 and Supplementary Table S4). Two types of ACSLs are reported to be responsible for the production of long-chain acyl-CoA, which can be utilized for cellular lipid synthesis or degradation via beta-oxidation, and different subcellular localizations can contribute to channel fatty acids toward different anabolic and catabolic pathways (Mashek et al., 2007). Two ACSLs can enhance storage lipid accumulation and import long-chain fatty acids from the extracellular environment in *P. tricornutum* (Guo et al., 2014). Another important enzyme, ACACA, which catalyzes the first committed step of the fatty acid synthetic pathway, was also identified to be down-regulated in the N-, P-, and Si-deficient cells (Figure 8 and Supplementary Table S4). The down-regulation of ACACA was verified in mRNA level (Figure 7), which was inconsistent with the result after 24 h of Si starvation reported by Smith et al. (2016). The change of this gene in our study could be considered as a delayed response to Si deficiency. Repression of this enzyme is associated with the long-chain acyl-CoA that is utilized for lipid synthesis, but is not degraded via beta-oxidation (Kamiryo et al., 1979). In addition, our study showed that the total lipid content per cell increased in the N- and Si-deficient cells but altered insignificantly in the P-deficient cells (Figure 2). Total lipids can be divided into neutral lipids such as triacylglycerides, and polar lipids such as phospholipids. Previous studies indicate that N, P, or Si starvation can increase the cellular neutral lipid content in *T. pseudonana*, as well as other diatom and green algal species (Jiang et al., 2012; Gong et al., 2013; Smith et al., 2016; Hunter et al., 2018). We presume that unchanged total lipid content in the P-deficient cells may result from a great deal of degradation of phospholipids. Thus, these results indicated that the increasing lipid synthesis of *T. pseudonana* in response to different macronutrient deficiencies might be caused by utilization of long-chain fatty acids via ACSL rather than *de novo* fatty acid synthesis.

Carbohydrate contents increased in the N-, P-, and Si-deficient cells, especially in the P-deficient cells (Figure 2). P deficiency can generate amounts of carbon intermediates from the degradation of phospholipid, which are further transferred to carbohydrate through glycolysis and gluconeogenesis. Expectably, most of the DEPs involved in glycolysis and gluconeogenesis were up-regulated in the P-deficient cells (Figures 5C, 8B). These results suggested that N, P, or Si deficiency enhanced the accumulations of carbohydrates and neutral lipids.

Cellular Si content decreased in all macronutrient deficient cells. Polyamine is one major silica polymerization component (Kröger et al., 2000), which is rich in N, and decrease of cellular Si content in the N- deficient cells might be related to decrease of polyamine synthesis. On the other hand, the decrease of cellular Si content in the P-deficient cells might be caused by energy limitation or alteration of the plasma membrane structure.

## Conclusion

Our study showed that deficiencies of different macronutrients resulted in both common and specific responses. Macronutrient deficiencies depressed carbon fixation and photorespiration but promoted neutral lipid and carbohydrate accumulation. In addition, the proteins involved in nutrient transport and utilization were all up-regulated in each individual macronutrient deficient cells, and utilization of organic N or P was enhanced. In both N- and P-deficient cells, photosynthesis, chlorophyll biosynthesis, carbon fixation and protein biosynthesis were down-regulated, which resulted in the reduction of chlorophyll a, protein and cellular N content, while carbohydrate metabolism was up-regulated. Moreover, intranuclear processes were exclusively up-regulated in the N-deficient cells, resulting in the increase of cellular P. However, the Si-deficient cells exhibited a significantly different response compared with the N- or P-deficient cells: LHC proteins, chloroplastic ATP synthase, as well as transcription and protein synthesis in the chloroplast were up-regulated, which increased the contents of chlorophyll a, cellular N and P. Overall, our study provided new insights into both the common and specific responses of *T. pseudonana* to different macronutrient deficiencies, and identified specific proteins potentially indicating ambient individual macronutrient deficiency. Future work should be devoted to the responses of different diatom species to different macronutrient deficiencies to unveil universality and specificity among diatoms.

## Data Availability

The raw mass spectrometry proteomic data and analysis files have been submitted to ProteomeXchange via PRIDE database (www.ebi.ac.uk/pride/archive/) with identifier PXD011133.

## Author Contributions

D-ZW and X-HC designed the research. X-HC, Y-YL, and J-LL performed the experiments. X-HC, Y-YL, and D-ZW performed analysis of the data and the interpretation of the paper with significant contributions by HZ and Z-XX. X-HC, D-ZW, and Y-YL wrote the article. LL contributed to the analytic instruments.

## Conflict of Interest Statement

The authors declare that the research was conducted in the absence of any commercial or financial relationships that could be construed as a potential conflict of interest.

## Footnotes

1 http://www.ncbi.nlm.nih.gov/RefSeq/
