# Supplementary material for: Quantitative Proteomics Reveals Common and Specific Responses of a Marine Diatom Thalassiosira pseudonana to Different Macronutrient Deficiencies
Source: Front Microbiol. 2018 Nov 14;9:2761. doi: 10.3389/fmicb.2018.02761 (PMC6246746; doi:10.3389/fmicb.2018.02761)
Supplement: Supplementary file 1 [file Table_1.DOCX]

Table S1. Primers used in this study.

| Biological processes | Gene name | Protein accession number | Protein gi number | Primer sequences |
| --- | --- | --- | --- | --- |
| N transport and utilization | Nitrate/nitrite transporter (NRT1) | XP_002288802 | gi\|223998258 | F: GGTGTCACCCAGCTCGTTAT  R: TCCTGCAATCATTCCAACAA |
|  | Nitrate/nitrite transporter (NRT2) | XP_002295904 | gi\|224004506 | F: GGTGTCACCCAGCTCGTTAT  R: TACCAACAATGGCAGGAACA |
|  | Urea-proton symporter (DUR3) | XP_002292926 | gi\|224007933 | F: AAAGACCGCCCATCTTACCT  R: ACACCCGTAAGTGCCTCAAC |
|  | Xanthine/uracil permease (TC.NCS2) | XP_002295239 | gi\|224013174 | F: CACGACTTTCAGCCAGAACA  R: CACGTTGGCAAACAAGAAGA |
|  | Urease(URE) | XP_002296690 | gi\|224014054 | F: AGCTTATGGTTTGCCACCAC  R: AGAGAGTGCACCGAGATCGT |
| P transport and utilization | Sodium phosphate co-transporter(SLC34A) | XP_002292964 | gi\|224008010 | F: TTCACGTGCTTCTGATTTGC  R: AGGACGGTCTTTACGGGACT |
|  | Alkaline phosphatase (phoD) | XP_002294783 | gi\|224012260 | F: GACGTTGGGTTATGCTCGTT  R: GTCGACTCTTCAGCCGAATC |
|  | Alkaline phosphatase (phoA) | XP_002286339 | gi\|223993311 | F: GACGTTGGGTTATGCTCGTT  R: GTCGACTCTTCAGCCGAATC |
|  | Alkaline phosphatase (AP) | XP_002286092 | gi\|223992817 | F: TCTGGAGTTCCAATCCCTTG  R: GCTGGCTGGCTTGAAGTATC |
|  | 5'-nucleotidase/UDP-sugar diphosphatase (ushA) | XP_002295546 | gi\|224011543 | F: GACTACCGACGCCTTCACTC  R: TATTCGATGAATCGCCTTCC |
|  | Glycerophosphoryl diester phosphodiesterase (glpQ） | XP_002292125 | gi\|224006329 | F: AGGCAGTAGCACTCGACGAT  R: AAGCATCCACATTGGAGGAG |
|  | Vacuolar transporter chaperone 4 (VTC4) | XP_002295322 | gi\|224013341 | F: AACGAGCGTACCTTCCTTCA  R: GTGCAATGCATAGAGGCAGA |
| Si transport | Silicic acid transporter (SIT1) | XP_002290700 | gi\|82527193\| | F: CATGGCTGTTGTTGGTATGC  R: TGCGTCCGAAGAAAGAAGTT |
|  | Silicic acid transporter (SIT2) | XP_002295920 | gi\|82527195\| | F: TGAGCGTGGAACTTCCTTCT  R: TGGTAAGGGAGGTGAACGAC |
| Carbon fixation | Pyruvate phosphate dikinase, chloroplastic (PPDK) | XP_002290738 | gi\|224002132 | F: GAAGACTGCCGAGGAACTTG  R: GTGAACTCGGGTTTGACGAT |
|  | Delta carbonic anhydrase (CA) | XP_002290131 | gi\|589908182 | F: CTTGATTGATGCCGATGATG  R: TCAACTGTCTCCTCGCACAC |
| Photorespiration | Glycine decarboxylase t-protein (gcvT) | XP_002292225 | gi\|224006530 | F: GCAAGGAAACGTGTCGGTAT  R: GCCTTTGCCAACTCAGTCTC |
| Glycolysis | 6-phosphofructokinase (pfkA) | XP_002286176 | gi\|223992985 | F: AAACTCATGGGTCGTTCCAG  R: GTTAGGACCGTCCAAGACGA |
|  | Pyruvate kinase PYK2 | XP_002289405 | gi\|223999465 | F: ACGGTTGTTTCGTGTGATGA  R: GTCGTGCTTGATACCCCAGT |
|  | Pyruvate kinase PYK1 | XP_002289867 | gi\|224000389 | F: GGAGAGGTTGTGTGCCGTAT  R: GGCGATGTAATCAACCTCGT |
| Pyruvate metabolism | Pyruvate dehydrogenase E1 component subunit alpha−1 (PDHA1) | XP_002287523 | gi\|223995699 | F: GGAGGGTCGATGCATTTTTA  R: ATGATTAGCAAACGCCAACC |
|  | Pyruvate dehydrogenase E1 component subunit beta−1 (PDHB1) | XP_002296440 | gi\|224013552 | F: GGTGGTTTTGGAGCATGAGT  R: TCCCCTCCCTCTCAATCTTT |
| TCA cycle | Citrate synthase (CS) | XP_002294538 | gi\|224011770 | F: CCATCATCCCTGAGATTGCT  R: TAGTGTGAGCCGAAGCATTG |
|  | Isocitrate dehydrogenase (icd) | XP_002288203 | gi\|223997060 | F: TCTTGTCGGTGTCTCTGACG  R: CGTATTCCAACTGAGCAGCA |
| Chlorophyll biosynthesis | Chlorophyll synthase (chlG) | XP_002289474 | gi\|223999603 | F: TCTTTGGTTCTTGGGTTTCG  R: GGACGATCCAACTCTCCAAA |
| Lipid biosynthesis | Acetyl-CoA carboxylase (ACACA) | XP_002287470 | gi\|223995593 | F: TGGTGCTCACGTCAAGAAAG  R: CTCGGGATTCTCCAATTCAA |
|  | Long-chain acyl-CoA synthetase (ACSL) | XP_002293417.1 | gi\|224008917 | F:ACGGCTCAGGCTACAGACAT  R: CAAAACGTGGCGATATCCTT |
