# Supplementary material for: Quantitative Proteomics Reveals Common and Specific Responses of a Marine Diatom Thalassiosira pseudonana to Different Macronutrient Deficiencies
Source: Front Microbiol. 2018 Nov 14;9:2761. doi: 10.3389/fmicb.2018.02761 (PMC6246746; doi:10.3389/fmicb.2018.02761)
Supplement: Supplementary file 2 [file Table_2.DOCX]

Table S2. Contents of cellular elements and biosynthetic compounds normalized to mean cell volume of *T. pseudonana* under different macronutrient deficient conditions. “0.0000” means a P value of less than 0.0001. **P < 0.01 and *P < 0.05 indicate significant correlation.

| Contents per volume (fg/μm3) | Control  (mean ± S.D.) | -N  (mean ± S.D.) | P value  (-N vs Control) | -P  (mean ± S.D.) | P value  (-P vs Control) | -Si  (mean ± S.D.) | P value  (-Si vs Control) |
| --- | --- | --- | --- | --- | --- | --- | --- |
| Cellular C | 185.05±8.85 | 163.54±8.82 | 0.0406* | 254.85±26.74 | 0.0207* | 230.49±45.37 | 0.1643 |
| Cellular N | 28.95±0.17 | 5.00±1.02 | 0.0000** | 13.50±0.32 | 0.0000** | 27.17±4.95 | 0.5441 |
| Cellular P | 5.15±0.43 | 12.54±0.87 | 0.0017** | 0.35±0.03 | 0.0002** | 18.31±2.41 | 0.0054** |
| Cellular Si | 63.82±5.91 | 24.00±0.55 | 0.0109* | 19.04±2.52 | 0.0101* | 18.03±1.87 | 0.0009** |
| Chl. a | 6.36±0.58 | 1.41±0.15 | 0.0001** | 2.38±0.10 | 0.0003** | 7.56±0.95 | 0.0280* |
| Carbohydrate | 56.34±7.59 | 239.29±13.93 | 0.0000** | 364.46±32.94 | 0.0001** | 74.34±18.96 | 0.0207* |
| Protein | 131.93±9.11 | 46.46±4.87 | 0.0001** | 59.45±3.29 | 0.0002** | 116.70±21.53 | 0.3224 |
| Lipid | 1451.01±138.08 | 2553.59±102.59 | 0.0004** | 1136.47±121.00 | 0.0412* | 1694.28±208.93 | 0.1678 |
