# Supplementary material for: Quantitative Proteomics Reveals Common and Specific Responses of a Marine Diatom Thalassiosira pseudonana to Different Macronutrient Deficiencies
Source: Front Microbiol. 2018 Nov 14;9:2761. doi: 10.3389/fmicb.2018.02761 (PMC6246746; doi:10.3389/fmicb.2018.02761)
Supplement: Supplementary file 5 [file Table_5.DOCX]

Table S5. The significantly enriched KEGG pathways in *T. pseudonana* under different macronutrient deficiencies (P-value of less than 0.05).

| KEGG Pathway | Differential expressed protein number | P-value |
| --- | --- | --- |
| N deficiency (-N vs Control) |  |  |
| Photosynthesis-antenna proteins | 23 | 1.4376E-13 |
| Photosynthesis | 17 | 2.1266E-07 |
| Ribosome | 23 | 2.6902E-02 |
| Porphyrin and chlorophyll metabolism | 15 | 1.1392E-05 |
| Terpenoid backbone biosynthesis | 7 | 3.3413E-02 |
| Glycine, serine and threonine metabolism | 12 | 5.7801E-03 |
| Phenylalanine, tyrosine and tryptophan biosynthesis | 7 | 4.7789E-02 |
| Glyoxylate and dicarboxylate metabolism | 14 | 5.5942E-05 |
| Pyruvate metabolism | 12 | 2.7822E-02 |
| Alanine, aspartate and glutamate metabolism | 10 | 8.7791E-03 |
| Propanoate metabolism | 9 | 1.7656E-02 |
| Carbon fixation pathways in prokaryotes | 7 | 3.0654E-03 |
| Valine, leucine and isoleucine biosynthesis | 5 | 3.2721E-02 |
| Nitrogen metabolism | 8 | 1.2459E-03 |
| TCA cycle | 9 | 2.9626E-02 |
| Ribosome biogenesis in eukaryotes | 13 | 8.5562E-03 |
| P deficiency (-P vs Control) |  |  |
| Ribosome | 89 | 1.3438E-55 |
| Photosynthesis-antenna proteins | 14 | 2.6700E-05 |
| Porphyrin and chlorophyll metabolism | 16 | 2.0146E-06 |
| Photosynthesis | 9 | 2.4769E-02 |
| Glyoxylate and dicarboxylate metabolism | 9 | 2.9195E-02 |
| Glycine, serine and threonine metabolism | 11 | 1.5565E-02 |
| Carbon fixation pathways in prokaryotes | 6 | 1.4008E-02 |
| Pyruvate metabolism | 13 | 1.1473E-02 |
| Carbon fixation in photosynthetic organisms | 11 | 7.7069E-03 |
| Two-component system | 5 | 4.1593E-02 |
| Glycerophospholipid metabolism | 9 | 3.9648E-02 |
| Pentose phosphate pathway | 12 | 3.1021E-03 |
| Glycolysis/Gluconeogenesis | 17 | 1.6804E-02 |
| Si deficiency (-Si vs Control) |  |  |
| One carbon pool by folate | 7 | 1.3509E-04 |
| Carbon fixation pathways in prokaryotes | 5 | 6.9040E-03 |
| Selenocompound metabolism | 4 | 1.4090E-02 |
| Glyoxylate and dicarboxylate metabolism | 7 | 1.5763E-02 |
| Glycine, serine and threonine metabolism | 10 | 1.1289E-03 |
| Nitrogen metabolism | 5 | 1.1115E-02 |
| Amino sugar and nucleotide sugar metabolism | 8 | 4.6007E-02 |
| Porphyrin and chlorophyll metabolism | 9 | 1.0409E-03 |
| ECM-receptor interaction | 2 | 2.7404E-02 |
| Photosynthesis | 6 | 4.3675E-02 |
